# A critically ill patient after a colchicine overdose below the lethal dose: a case report

**DOI:** 10.1186/s13256-018-1737-5

**Published:** 2018-07-04

**Authors:** Ichiro Hirayama, Takahiro Hiruma, Yoshihiro Ueda, Kent Doi, Naoto Morimura

**Affiliations:** Department of Acute Medicine, The University of Tokyo Hospital, 7-3-1 Hongo, Bunkyo-ku, Tokyo, 113-8655 Japan

**Keywords:** Intestinal Behçet’s disease, Colchicine, Drug overdose, Lethal dose 50, Multiple organ failure

## Abstract

**Background:**

Although 0.8 mg/kg is considered a lethal dose of colchicine, fatal cases of patients who followed a critical disease course after an intake below this lethal dose have been reported.

**Case presentation:**

An 18-year-old Japanese woman who had taken an overdose of prescription colchicine (15 mg; 0.2 mg/kg) was brought to our emergency out-patient department. Although her colchicine intake was below 0.8 mg/kg (considered the lethal dose), she reached a critical state and underwent three phases characterizing colchicine poisoning (gastrointestinal symptoms, multiple organ failure, and recovery). Her condition was critical, with a Sequential Organ Failure Assessment score of a maximum of 14.

**Conclusions:**

Patients might reach a critical stage after colchicine ingestion at a non-lethal dose. Thus, it might be necessary to review which dose of colchicine should be considered lethal.

## Background

Colchicine is a therapeutic drug for intestinal Behçet’s disease. Although the lethal dose of colchicine is considered to be 0.8 mg/kg, fatal cases of patients who followed a critical disease course after an intake below the lethal dose of colchicine have been reported [1]. It has been shown that colchicine doses between 7 and 26 mg, which are below the lethal level, have resulted in death [2–6].

Here, we report the case of a patient who was prescribed with colchicine for pain relief. Ingestion of an overdose of 15 mg (0.2 mg/kg) resulted in multiple organ failure.

A patient who ingests colchicine could reach a critical stage, despite an intake below the lethal level. This is believed to be due to interaction of colchicine with other drugs. However, this patient was not administered any other drug that might interact with colchicine.

## Case presentation

An 18-year-old Japanese woman, with a history of intestinal Behçet’s disease, complained of abdominal pain on the day she was brought to our hospital. She took prescription colchicine at a dose of 15 mg (30 tablets 0.5 mg each), which is equivalent to 0.2 mg/kg. As her condition did not improve, she was brought to the emergency department. She had a past medical history of fibromyalgia, in addition to intestinal Behçet’s disease. Prior to admission, she was taking Neurotropin® (non-protein extract isolated from the inflamed skin of rabbits inoculated with vaccinia virus), pregabalin, butylscopolamine bromide, Lactomin (lactic acid bacteria – *Lactobacillus acidophilus*, *Bifidobacterium longum*), and colchicine. She neither smoked tobacco nor drank alcohol. Her social and environmental history was unremarkable. She had never worked. Her mother had schizophrenia.

Her vital signs on arrival included blood pressure (BP) of 128/90 mmHg, pulse of 102 beats per minute, regular respiration rate of 18 breaths per minute, blood oxygen saturation (SpO_2_) of 98% room air, and body temperature of 37.5 °C; she was alert and conscious. She had upper abdominal tenderness with no rebound tenderness. The results of her cardiac, pulmonary, and neurological examinations were unremarkable. Complete blood count, renal function tests, urine analysis, and bacteria tests were normal except for mild hepatic dysfunction and elevated d-dimer levels (Table 1). Although colchicine was administered below the lethal dose, she was admitted for observation.

**Table 1:** Laboratory findings, blood culture, and urine analysis at arrival

Laboratory data			Other examinations	
White blood cells	3500	/mm^3^	Blood culture	Not detected
Hemoglobin	12.0	g/dl	Urine analysis	Normal
Platelet	26.9 × 10^4^	/mm^3^		
AST	69	U/l		
ALT	49	U/l		
γ-GT	83	U/l		
Total bilirubin	0.3	mg/dl		
Amylase	99	U/l		
BUN	5.6	mg/dl		
Creatinine	0.50	mg/dl		
CK	81	U/l		
Na	140	mEq/l		
K	4.0	mEq/l		
D-dimer	10.7	μg/ml		

*ALT* alanine aminotransferase, *AST* aspartate aminotransferase, *BUN* blood urea nitrogen, *CK* creatine kinase, *γ-GT* γ-glutamyltransferase, *K* potassium, *Na* sodium

On the day after the hospital admission, she developed acute respiratory distress syndrome (ARDS), thus, tracheal intubation using an artificial ventilator was performed. She also became dehydrated, due to diarrhea, which developed after admission. Peripheral circulatory insufficiency gradually worsened, and large amounts of infusion loads (including blood transfusions) were unable to maintain her BP. Her lactic acid level reached a maximum of 19 mmol/L. At maximum disease severity, she required noradrenaline 1 μg/kg/minute, vasopressin 2 units/hour, steroids 200 mg/day, dopamine 10 μg/kg/minute, dobutamine 4 μg/kg/minute, and adrenaline 0.15 μg/kg/minute to maintain BP. She also showed other organ failures, including worsening hepatic dysfunction after admission with maximum aspartate aminotransferase (AST) level of 16,520 U/L as well as kidney dysfunction with associated anuria. Continuous renal replacement therapy was initiated for anuria and metabolic acidosis. At 36 hours post-admission, her lactic acid level reached its peak, and her hemodynamic reached a level at which she could tolerate water removal. At 72 hours post-admission, she was administered with granulocyte colony-stimulating factor due to the appearance of myelosuppression. In addition to the broad-spectrum antimicrobial administration, she was commenced on a course of antimycotic medication. Table 2 shows the laboratory results of organs function over time.

**Table 2:** Trend in the laboratory results over time

Laboratory data	Unit	0 hr	12 hr	24 hr	36 hr	48 hr	72 hr	96 hr	120 hr
White blood cells	× 10^3^/mm^3^	3.5	19.9	19.4	9.4	5.6	2.2	0.8	0.9
Platelets	×10^3^/mm^3^	26.9	17.2	9.9	2.6	3.8	2.8	3.2	3.0
AST	U/l	69	153	161	4190	13,000	16,520	9050	3630
ALT	U/l	49	63	55	2635	4392	4994	2904	1704
Total bilirubin	mg/dl	0.3	0.4	0.6	1.4	1.7	2.8	5.5	7.8
CK	U/l	81	117	221	1341	5401	43,140	149,450	100,050
Creatinine	mg/dl	0.50	0.58	0.86	1.23	1.34	1.21	0.95	1.15
Lactic acid	mmol/l	1.6	3.6	11.2	17.0	14.0	9.3	5.5	6.4

*ALT* alanine aminotransferase, *AST* aspartate aminotransferase, *CK* creatine kinase

**Fig. 1:**
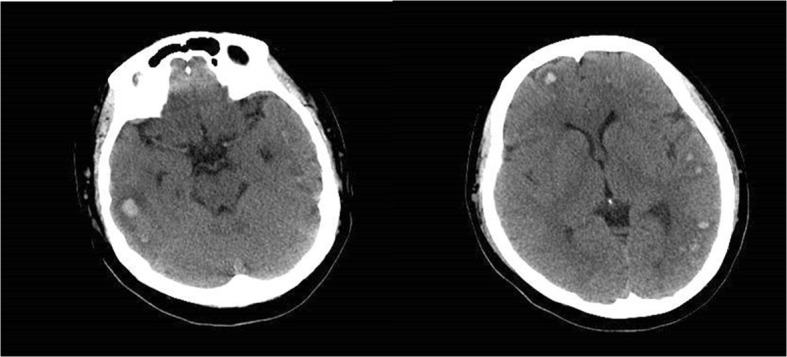
Plain head computed tomography conducted on day 7 of the patient’s hospital stay showing multiple intracranial hemorrhages

Approximately 1 week after admission, her BP could be maintained without the use of vasopressor drugs; however, as she showed poor alert wakefulness, a head computed tomography was performed. The results showed multiple intracranial hemorrhages of up to 12 mm in diameter (Fig. 1), thought to be associated with disseminated intravascular coagulation (DIC). As the disease course progressed, the hematomas were absorbed and our patient was able to respond to verbal commands. Approximately 2 weeks post-admission, she presented with marked hair loss.

**Fig. 2:**
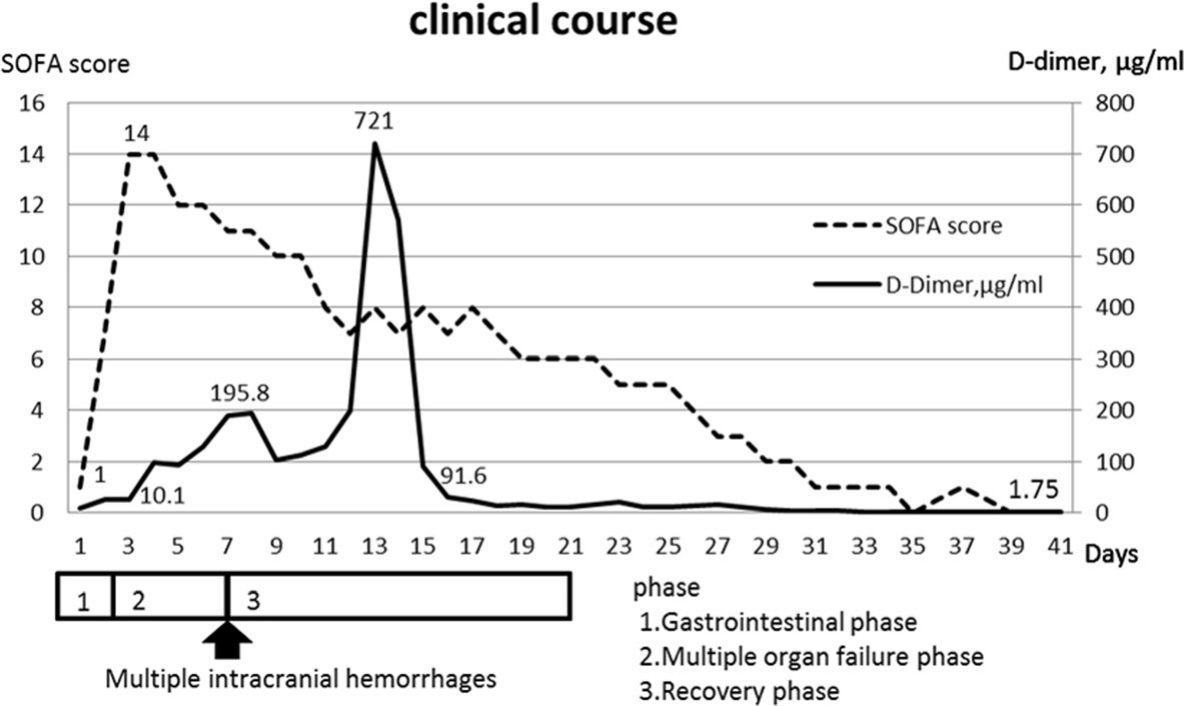
Post-admission clinical course. On the day following admission, the patient presented with multiple organ failure and a markedly elevated Sequential Organ Failure Assessment score. After going through the three phases, her organ failure had improved by the time of discharge. *SOFA* Sequential Organ Failure Assessment

Subsequently, her general condition improved and, at discharge, all organ insufficiency had improved. The progress of her Sequential Organ Failure Assessment (SOFA) scores and D-dimer levels are shown in Fig. 2. She worked hard at rehabilitation. By 6 months after discharge, she had no disorders and had resumed normal life as before her hospitalization. Table 3 shows the blood tests at follow-up.

**Table 3:** Laboratory findings at follow-up

Laboratory data		
White blood cells	7900	/mm^3^
Hemoglobin	12.7	g/dl
Platelet	29.2 × 10^4^	/mm^3^
AST	15	U/l
ALT	18	U/l
γ-GT	60	U/l
Total bilirubin	0.6	mg/dl
Amylase	107	U/l
BUN	10.3	mg/dl
Creatinine	0.46	mg/dl
CK	56	U/l
Na	140	mEq/l
K	3.9	mEq/l
D-dimer	1.9	μg/ml

*ALT* alanine aminotransferase, *AST* aspartate aminotransferase, *BUN* blood urea nitrogen, *CK* creatine kinase, *γ-GT* γ-glutamyltransferase, *K* potassium, *Na* sodium

## Discussion

An 18-year-old woman who was prescribed colchicine used the drug at a dose of 15 mg (0.2 mg/kg). Despite the non-lethal dose, the colchicine intake led to multiple organ failure. Unlike what is known from a previous report, this patient reached a critical stage in her presentation, which was not related to drug interaction.

Colchicine is a therapeutic drug used in the treatment of intestinal Behçet’s disease. Fatal cases, in patients whose disease progressed to a critical stage, despite colchicine intake below the lethal dose have been reported. Patients sometimes overdose on colchicine when it is prescribed for pain relief; this indicates the need for caution when prescribing this drug. Although the lethal colchicine dose is considered to be 0.8 mg/kg, the patient in the present case reached a critical stage after an intake of only 0.2 mg/kg.

Two main conclusions can be derived from this case. First, patients can follow a critical disease course that can be fatal, even after ingestion of a colchicine dose that is below the lethal level. Second, patients can reach the critical stage without a clear cause, after the intake of colchicine below the lethal level.

The symptoms of colchicine poisoning progress through a three-phase clinical course as follows. First (within 24 hours of ingestion), the patient presents with severe gastrointestinal symptoms, including vomiting, diarrhea, and abdominal pain. Second, the patient develops ARDS and myelosuppression between the second and seventh day of ingestion. This then leads to multiple organ failure, including liver and kidney failure. Finally, temporary hair loss was seen after the seventh day of ingestion (during the recovery phase) [1, 7]. We believe that the progress of the present case is consistent with colchicine poisoning. Our patient also experienced multiple intracranial hemorrhages thought to be associated with DIC caused by the gastrointestinal symptoms and multiple organ failure.

Other reports of fatal cases after colchicine ingestion at non-lethal levels discussed the possibility that interaction with other drugs might have caused the patients to become critical. In the present case, the patient was not administered with any other drug (such as cytochrome P450 3A4 inhibitors) that might have interacted with colchicine; therefore, it remains unclear why she reached the critical stage.

Based on our findings, we believe that the LD50 standard for colchicine should be reviewed.

## Conclusions

Patients might become critical after colchicine ingestion at a dose below the lethal level. Although reports have suggested that interactions with other drugs might result in patients’ death, we believe that it might be necessary to review the fatal dose of colchicine. We learned from the presented case that patients on colchicine could reach a critical stage despite an intake below the lethal colchicine level; therefore, we intend to monitor the patient over time.

## Abbreviations

ARDS: Acute respiratory distress syndrome
AST: Aspartate aminotransferase
BP: Blood pressure
DIC: Disseminated intravascular coagulation
SOFA: Sequential Organ Failure Assessment
SpO_2_: Blood oxygen saturation
